# Pathology and Treatments of Alzheimer’s Disease Based on Considering Changes in Brain Energy Metabolism Due to Type 2 Diabetes

**DOI:** 10.3390/molecules29245936

**Published:** 2024-12-16

**Authors:** Hidekatsu Yanai, Hiroki Adachi, Mariko Hakoshima, Hisayuki Katsuyama

**Affiliations:** Department of Diabetes, Endocrinology and Metabolism, National Center for Global Health and Medicine Kohnodai Hospital, 1-7-1 Kohnodai, Ichikawa 272-8516, Chiba, Japan; dadachidm@hospk.ncgm.go.jp (H.A.); d-hakoshima@hospk.ncgm.go.jp (M.H.); d-katsuyama@hospk.ncgm.go.jp (H.K.)

**Keywords:** Alzheimer’s disease, amyloid beta, glucagon-like peptide 1 receptor agonists, insulin resistance, oxidative stress, type 2 diabetes

## Abstract

Alzheimer’s disease (AD) is a progressive neurodegenerative disorder with cognitive dysfunction, memory decline, and behavioral disturbance, and it is pathologically characterized by the accumulation of amyloid plaques and neurofibrillary tangles in the brain. Although various hypotheses have been proposed to explain the pathogenesis of AD, including the amyloid beta hypothesis, oxidative stress hypothesis, and abnormal phosphorylation of tau proteins, the exact pathogenic mechanisms underlying AD remain largely undefined. Furthermore, effective curative treatments are very limited. Epidemiologic studies provide convincing evidence for a significant association between type 2 diabetes and AD. Here, we showed energy metabolism using glucose, lactate, ketone bodies, and lipids as energy substrates in a normal brain, and changes in such energy metabolism due to type 2 diabetes. We also showed the influences of such altered energy metabolism due to type 2 diabetes on the pathology of AD. Furthermore, we comprehensively searched for risk factors related with type 2 diabetes for AD and showed possible therapeutic interventions based on considering risk factors and altered brain energy metabolism due to type 2 diabetes for the development of AD.

## 1. Introduction

Alzheimer’s disease (AD) is the leading cause of cognitive impairment in older individuals and one of the key factors of disability and mortality in later life throughout the world [1]. AD is a progressive neurodegenerative disorder characterized by the accumulation of amyloid plaques and neurofibrillary tangles in the brain, and this disease is an incurably neurodegenerative disorder with cognitive dysfunction, memory decline, and behavioral disturbance [2]. Various hypotheses have been proposed to explain the pathogenesis of AD, including the amyloid beta (Aβ) hypothesis [3], oxidative stress hypothesis [4], and abnormal phosphorylation of tau proteins [5]. However, the exact pathogenic mechanisms underlying AD remain largely undefined. The accumulation of soluble and insoluble aggregated Aβ may initiate or potentiate pathologic processes in AD. Aβ vaccines that are significantly capable of reducing amyloid plaques were unable to prevent AD progression [6]. Unfortunately, to date, almost all the clinical trials targeting the elimination of Aβ deposits have accomplished little to improve cognitive function [6,7]. Recently, lecanemab, a humanized IgG1 monoclonal antibody that binds with high affinity to Aβ soluble protofibrils, was developed and tested to treat patients with early AD [8]. Lecanemab reduced brain amyloid burden in patients with early AD and resulted in moderately less decline in measures of cognition and mental function than a placebo after 18 months [8]. However, longer trials are warranted to determine the efficacy and safety of lecanemab in early AD. Furthermore, effective therapy based on tau pathology is lacking [9,10,11].

Observational studies have suggested a significant association between type 2 diabetes and the development of AD [12,13]. Here, we show energy metabolism using glucose, lactate, and ketone bodies and lipids as energy substrates in the normal brain and changes in such energy metabolism due to type 2 diabetes. We show the influences of such altered energy metabolism due to type 2 diabetes on the pathology of AD. Furthermore, we comprehensively search risk factors related to type 2 diabetes for AD and show possible therapeutic interventions based on considering risk factors and altered brain energy metabolism due to type 2 diabetes for the development of AD.

## 2. Glucose Metabolism and Insulin Signaling in Normal Brain

The basic unit of the nervous system is the neuron, which receives and integrates information to form complex circuits that generate adaptive responses [14]. Neurons are divided into cell bodies, axons, and dendrites. The cell body has a nucleus. Proteins that are necessary for cell activities are produced within the cell body. Axons are projection-like structures that extend from the cell body and send signals to the next neuron or effector. The insulin-independent glucose transporter 3 (GLUT3) is the major glucose transporter in neurons. The density and distribution of GLUT3 in axons, dendrites, and neuronal soma correlates with local cerebral energy demands [15]. Neurons also express GLUT1 [16]. Activation by insulin induces GLUT4 translocation to the neuron cell membrane via an AKT-dependent mechanism [17,18] and is thought to improve glucose flux into neurons during periods of high metabolic demand, such as during learning [19]. Neurons express insulin receptor substrate (IRS)1 and IRS2, which are enriched in presynaptic and postsynaptic compartments [20]. Insulin has many roles in neurons, which are mediated by signaling through its two major effector pathways: the insulin–IRS–AKT and mitogen-activated protein kinase (MAPK) pathways [21,22]. The insulin receptor (IR) is highly enriched in synapses [23] and has important effects on neurosynaptic functioning [24,25,26,27]. Furthermore, insulin has a crucial role in the development and maintenance of excitatory synapses [28] and has been shown to promote dendritic spine formation and excitatory synapse development [29]. Insulin promotes neuronal survival by inhibiting apoptosis [30]. Facilitating the functions of neurons is supported by astrocytes, oligodendrocytes, and microglia.

Astrocytes are a subtype of glial cells that comprise most cells in the human central nervous system (CNS). They perform metabolic, structural, homeostatic, and neuroprotective tasks such as clearing excess neurotransmitters, stabilizing and regulating the blood–brain barrier (BBB), and promoting synapse formation [31,32,33,34]. Glucose is transported across the BBB through GLUT1 expressed on endothelial cells. Astrocytes take up glucose via GLUT1 and can process glucose glycolytically and transport lactate to neurons as an alternative fuel source during hypoglycemia in a process known as the astrocyte–neuron lactate shuttle [35,36]. Astrocyte–neuron lactate transport is required for long-term memory formation [37]. Like hepatocytes, astrocytes have been known for a long time to have a glucose-containing energy reserve in the form of glycogen [38]. It seems clear that it is mobilized during periods of high neuronal activity through the action of various neuroactive substances and that an energy substrate arising from glycogenolysis is released by astrocytes, most likely to be used by neurons. Unlike the situation in hepatocytes, however, this released energy substrate is not glucose but lactate. Astrocytes can export lactate through monocarboxylate transporter (MCT) 1/4 [39]. Lactate may be taken up by oligodendrocytes through MCT1 or by neurons through MCT2 [39]. Astrocytes bind insulin with high affinity and express IRS1, IRS2, and downstream signaling molecules AKT and MAPK [40]. The activation of such signaling is mediated by insulin or insulin-like growth factor 1 (IGF1), which also activates IRs [41,42,43]. Glial IRs are downregulated in response to chronically high levels of insulin, whereas neuronal IRs are not downregulated [44]. Astrocytes play a part in inflammatory responses in the brain, and insulin modulates astrocyte inflammatory cytokine secretion in response to inflammatory stimuli [41].

Oligodendrocytes form the myelin sheath which surrounds axons and enables rapid conduction of the nerve impulse [45]. They are the end product of a cell lineage which has to undergo a complex and precisely timed program of proliferation, migration, differentiation, and myelination to finally produce the insulating sheath of axons. GLUT1 exists in oligodendrocytes [46]. Glucose may be taken up by oligodendrocytes through GLUT1 [39]. The lactate from astrocytes may be taken up by oligodendrocytes through MCT1 [39]. The lactate is converted to pyruvate, which can be used for ATP production in oligodendrocytes and may be particularly important in producing lipids for myelination. Oligodendrocytes express IR, IRS1, and IRS2. AKT signaling is important for mediating oligodendrocyte proliferation, survival, differentiation, and myelination. The activation of AKT signaling by IGF1 in oligodendrocytes is well established and is known to promote differentiation and axonal ensheathment [47,48].

Microglia habitually interact with neuronal and non-neuronal elements, both structurally and functionally, in the healthy brain [49]. Microglia play an important role in the phagocytosis of synaptic structures during postnatal development, the phagocytosis of newborn neurons during adult neurogenesis, and the active remodeling of the peri-synaptic environment and the release of soluble factors in the mature and aging brain. Microglia predominantly express GLUT3 and GLUT5, but under inflammatory conditions, GLUT1 expression is upregulated to increase glucose uptake and promote glycolysis [50,51,52,53]. Microglia express IR and IRS1, and insulin modulates microglial inflammatory responses in a complex manner [40]. Microglia express MCT1, MCT2, and MCT4. MCT1 promotes classical microglial activation and a pro-inflammatory effect [54]. Enhanced cerebral expression of MCT1 and MCT2 was observed in activated microglial cells in a rat ischemia model [55]. Recently, the loss of microglial MCT4 has been reported to lead to defective synaptic pruning, which optimizes neural networks in the brain in mice [56].

Insulin is transported into the brain by IR-mediated transport [20]. GLUT1 and MCT1 transport glucose and ketone bodies across the BBB, respectively [57].

**Figure 1:**
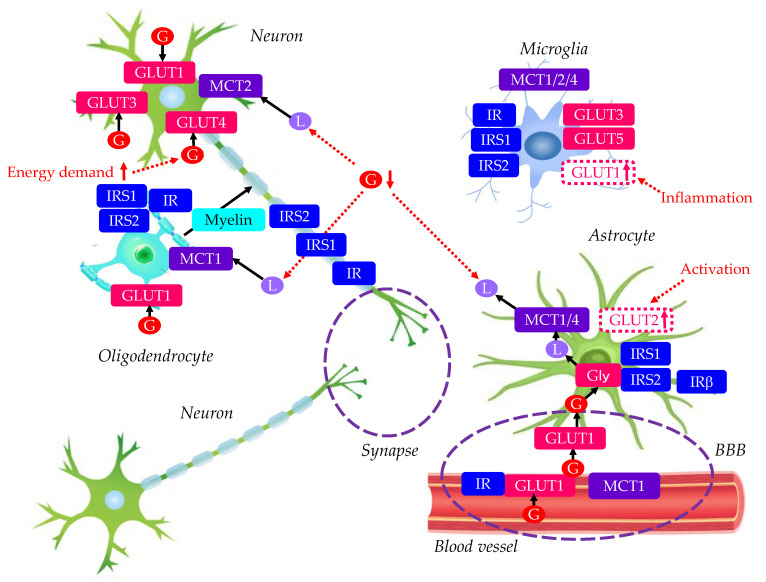
Glucose transporters, insulin receptor, insulin receptor substrate, and monocarboxylate transporters in normal brain. Red up and down arrows indicate increase and decrease in phenomenon, substances, and expression of molecules, respectively. Black arrows indicate the flow of substances. BBB, blood–brain barrier; G, glucose; GLUT, glucose transporter; Gly, glycogen; IR, insulin receptor; IRS, insulin receptor substrate; L, lactate; MCT, monocarboxylate transporter.

In summary, GLUTs, IR, IRS, and MCTs in normal brain are shown in Figure 1 and Table 1. All brain cells have IR and IRS, indicating the significance of insulin sensitivity in nervous integrity. Neurons have many GLUTs, and when energy demand increases, they increase the translocation of GLUT4 to obtain more energy. Furthermore, the energy demands of neurons are supported not only by glucose but also by lactic acid. Both glucose and lactic acid are supplied by astrocytes. The close cooperation between neurons and astrocytes may be crucial for a normal brain. Microglia also have many GLUTs and MCTs. Microglia may need a great amount of energy to maintain neurons by remyelination and synaptic pruning, which optimizes neural networks in the brain.

## 3. Lipid Metabolism in Normal Brain

The brain is a highly energy-demanding organ. The brain utilizes about 20% of the total O_2_ consumed by the resting body [58]. Lipids are components that make up over 50% of the brain. However, energy provision to the brain preferentially relies on a continuous circulatory supply of glucose to neural cells; the utilization of fatty acids (FAs) is less preferred [59]. About 20% of the total energy consumption of the brain originates from the oxidation of FAs [60,61], predominantly occurring in astrocytes and not neurons [60]. However, lipids are crucial in maintaining normal brain function as structural constituents of neuronal and neuroglial cell membranes and precursors of signaling molecules [62].

Neurons and astrocytes operate as a tightly coupled unit for energy metabolism in the brain. Furthermore, neuron–astrocyte metabolic coupling protects against activity-induced FA toxicity [63]. While neurons expend a considerable amount of ATP on neurotransmission, astrocytes provide neurons with metabolic substrates such as lactate and ketone bodies via MCTs, as well as antioxidants [64]. FAs are stored within cells as triglycerides (TGs) in lipid droplets in astrocytes. FAs in lipid droplets remove excess free FAs (FFAs) from the cytoplasm, which are toxic and disrupt mitochondrial membrane integrity [65,66]. Lipid droplets deliver FAs into mitochondria for an alternative energy source during periods of energy depletion [67]. Neurons do not make lipid droplets and have a low capacity for FA consumption in mitochondria for energy production [59]. Neurons are especially susceptible to reactive oxygen species (ROS) due to their poor antioxidative capacity [68]. Spurning the β-oxidation pathway in the mitochondria of neurons and the donation of metabolites to neurons for the synthesis of antioxidants by astrocytes protect neurons against FFA-linked lipotoxicity [68]. The neuronal autophagy of ROS-emitting mitochondria, combined with the transfer of degradation-committed FFAs for their disposal in astrocytes, is a potent protective strategy against ROS and the harmful activities of FFAs [68].

During enhanced neuronal activity, lactate produced by astrocytes is transported to neurons via MCTs. In neurons, lactate is used in oxidative metabolism for ATP production and/or for FA synthesis [69]. To avoid FFA toxicity, neurons release FAs in lipoprotein-like particles containing apo E, which can enter astrocytes by endocytosis. FAs are released from lipoprotein particles and are incorporated into lipid droplets [69]. FAs released from lipid droplets can be used as a fuel in mitochondrial β-oxidation in astrocytes. During energy deprivation, astrocytes metabolize FAs by β-oxidation to produce ketone bodies and transfer ketone bodies via MCTs to neuronal mitochondria for ATP synthesis [69].

Oligodendrocytes have much lower energetic demands than neurons, which require substantial energy to support the execution of action potentials [70,71]. Oligodendrocyte energy demands are required for myelination and providing metabolic support to neurons. Oligodendrocytes support the energetic needs of neurons, being responsible for supplying neurons with glycolytic products, lactate and pyruvate, providing extra fuel to maintain their intense activity [72,73,74]. FAs can be taken up from the blood by endothelial cells and passed through the BBB by astrocytes to oligodendrocytes. Both oligodendrocytes and oligodendrocyte precursor cells express fatty acid transport proteins (FATPs) and fatty acid binding protein (FABP) [75]. The ablation of these proteins impairs oligodendrocyte precursor cell proliferation and oligodendrocyte differentiation [76]. FAs derived from circulation can support myelination, although they cannot fully substitute specialized lipids that are internally synthesized by oligodendrocytes [77]. Interestingly, oligodendrocyte myelination also relies on lipids supplied by astrocytes, and when this synthesis is impaired, FAs derived by oligodendrocytes from circulation may compensate [78]. FAs are stored as lipid droplets, mainly in astrocytes, but also in oligodendrocytes during development; however, these stored lipids are mainly used for myelin production [69].

Microglia play an important role in the initiation, modulation, and resolution of inflammation [79,80]. Lipid metabolism in microglia is tightly regulated during development, damage, and disease [81]. Microglia promote primary myelination by the phagocytosis of apoptotic oligodendrocytes and myelin debris during early development [82,83,84,85]. The optimal remyelination after demyelination requires clearance of myelin-derived lipids such as ceramides, cholesterol, phospholipids, and sphingolipids by microglia [80,86,87]. Lipoprotein lipase (LPL) is predominantly expressed in the microglia [88,89]. LPL is crossly related to brain development, damage, and disease [90,91,92]. AD patients show a decrease in LPL activity in the hippocampus and cerebrospinal fluid (CSF) [92]. LPL plays an important role in supporting remyelination by facilitating lipid uptake and promoting FA oxidation while maintaining an anti-inflammatory microglial phenotype [86,91,93,94].

**Figure 2:**
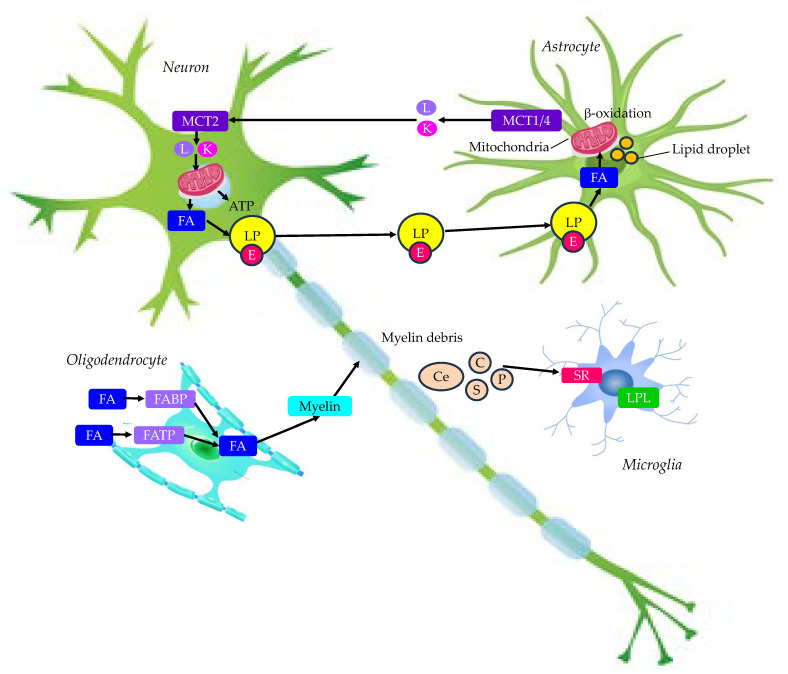
Lipid metabolism in normal brain. C, cholesterol; Ce, ceramide; E, apo E; FA, fatty acid; FABP, FA binding protein; FATP, FA transport proteins; GLUT, glucose transporter; K, ketone body; L, lactate; LP, lipoprotein-like particle; LPL, lipoprotein lipase; MCT, monocarboxylate transporter; P, phospholipid; S, sphingolipid; SR, scavenger receptor.

In summary, lipid metabolism in the normal brain is shown in Figure 2. Astrocytes store not only glycogen but also lipid droplets, which generate ketone bodies through the β-oxidation of FAs and provide them as an energy source for neurons. Such take-up of FAs by astrocytes protects neurons that are susceptible to FA toxicity from their toxicity. Oligodendrocytes require a lot of FAs to produce myelin and, therefore, have many transporters of FAs. Optimal remyelination after demyelination requires the clearance of myelin-derived lipids by microglia.

## 4. Risk Factors Related to Type 2 Diabetes for the Development of AD

The Rotterdam Study, a prospective population-based cohort study among 6370 elderly subjects, showed that diabetes almost doubled the risk of AD (relative risk (RR), 1.9; 95% confidence interval (CI), 1.2 to 3.1) [12]. The Hisayama study, a Japanese cohort study, showed that the age- and sex-adjusted incidence of AD was significantly higher in subjects with diabetes than in those with normal glucose tolerance. This association remained robust even after adjustment for confounding factors for AD (hazard ratio (HR), 2.05; 95% CI, 1.18 to 3.57; *p* = 0.01) [13]. These studies suggest that diabetes is significantly associated with the development of AD.

A meta-analysis showed that homeostatic model assessment of insulin resistance (HOMA-IR) was higher in AD patients than in controls (*p* < 0.001) [95], and HOMA-IR was negatively correlated with Mini-Mental State Examination (MMSE) scale scores (*p* = 0.001). Another meta-analysis showed an increased risk of AD in metabolic syndrome (HR, 1.10; 95% CI, 1.05 to 1.15) [96]. Insulin resistance is significantly associated with the development of AD. The expression of insulin and IGF-1/2 receptors was found to be markedly reduced in AD brains, which is correlated with pathological alterations, including increased glycogen synthase kinase-3 (GSK-3) activity and amyloid precursor protein (APP) mRNA levels [97]. Insulin/IGF-1 signaling defects impair the phosphatidyl-inositide 3-kinases (PI3K)/Akt pathway, producing harmful cascades in glucose metabolism [98]. The reduced expression and function of PI3K/Akt-mediated GLUTs in an AD brain could lead to brain glucose hypometabolism and a subsequent decline in mitochondrial ATP production [99]. A comparison of the function of the brain insulin–PI3K–Akt signaling pathway in the frontal cortices among subjects with AD, type 2 diabetes, type 2 diabetes and AD, and control subjects showed that the deficiency in the insulin–PI3K–Akt signaling was more significant in subjects with both type 2 diabetes and AD [98]. The levels and the activation of the insulin–PI3K–Akt signaling components were negatively correlated with the level of tau phosphorylation [98]. The MAPK pathway has been shown to be significantly activated in AD patients, which is correlated with increased neuroinflammation, tau hyperphosphorylation, and Aβ trafficking [99]. Tau hyperphosphorylation is related to excess activation of GSK-3β and MAPK, which are major tau kinases responsible for tau phosphorylation [100,101].

Diabetic dyslipidemia includes elevated TG and low-density lipoprotein cholesterol (LDL-C), as well as reduced high-density lipoprotein-C (HDL-C) [102]. A dose–response meta-analysis of a prospective cohort studies showed that every 3-mmol/L increase in serum TG is linearly associated with a 12% increase in the RR of AD [103]. LDL-C was associated with all measures of AD neuropathology and cerebral amyloid angiopathy independent of Apo E after adjusting for age, sex, cholesterol-lowering medication use, body mass index, smoking, and education [104]. A meta-analysis indicated that decreased HDL-C and increased LDL-C levels are related with an elevated risk of AD in subjects aged 60–70 [105,106]. In the meta-analysis, compared to healthy controls, AD subjects had a lower serum apo A-I (−0.31 g/L vs. controls, *p* < 0.0001), which is a major apoprotein of HDL [107].

Type 2 diabetes induces advanced glycation end product (AGE) accumulation in the brain, which leads to AD. AGEs are a crucial contributing factor of the onset and development of AD [108]. AGEs induce oxidative stress, inflammation, amyloid plaques, and neurofibrillary tangles [108]. The interaction of receptors for AGEs (RAGEs) with Aβ induces the amplification of oxidative stress, mitochondrial dysfunction, and inflammation, resulting in RAGE-induced neuronal pathophysiological changes and contributing to the development of AD [109].

Hyperglycemic-induced oxidative stress and inflammation are closely associated with the development and progression of type 2 diabetes and its complications [110]. Abnormal oxidative stress is an established feature of AD. A meta-analysis of in vivo magnetic resonance spectroscopy studies showed that neuroinflammation and oxidative stress may occur in the early stages of AD, and may likely precede neuron loss in its progression [111]. Isoprostanes are biomarkers of oxidative stress. A systematic review of observational studies on the associations of F2-isoprostanes and the specific biomarker 8-iso-prostaglandin F2α, which is the most abundantly produced F2-isoprostane, with AD was conducted [112]. F2-isoprostane levels were significantly associated with the development of AD [112]. F2-isoprostane and 8-iso-prostaglandin F2α levels were significantly elevated in tissue samples of the frontal lobe of AD patients. Moreover, F2-isoprostane levels in the CSF and 8-iso-prostaglandin F2α levels in blood samples of AD patients were significantly increased. The markers of lipid peroxidation are elevated in blood in AD, and total antioxidant capacity is decreased [113]. Non-enzymatic antioxidants in blood (uric acid; vitamins A, E, and C; α- and β-carotene) were significantly decreased in AD patients [114].

The 18-kDa translocator protein is increasingly recognized as a molecular target for positron emission tomography (PET) imaging of inflammatory responses in various CNS disorders [115]. Regions known to be vulnerable in AD, such as the posterior cingulate cortex and hippocampus, are consistently shown to display increased translocator protein PET signals [116]. In fact, volume changes assessed on magnetic resonance imaging (MRI) in these two regions, designated as epicenters of the pathology, are considered the best predictors of AD progression [117].

Mitochondrial dysfunction has been broadly described in the early stages of both type 2 diabetes and AD, and this dysfunction has emerged as a plausible molecular link between them [118]. Mitochondrial dysfunction has been observed prior to amyloid plaque deposition [119]. Mitochondrial dysfunction induces the overproduction of ROS [120,121]. In AD, mitochondrial abnormalities are considered the main source of oxidative stress [122]. PET studies have also indicated that mitochondrial dysfunction is shown in the early stage of AD, and mitochondrial-related energy failure may precede glycolysis-related hypometabolism in regions with pathologically confirmed early neurodegeneration in AD [123]. Proteomics studies have shown that mitochondrial dysfunction is an early biochemical event that might play a central role in driving AD pathogenesis [124].

Autophagy is a self-cleaning and self-eating effect [125]. Autophagy maintains cellular homeostasis by eliminating damaged organelles and misfolded proteins [126]. Autophagy exerts a protective effect on pancreatic β-cells [127]. A prolonged high-glucose–high-fat diet has been reported to inactivate autophagy [128]. Autophagy plays a crucial role in maintaining normal islet structure. In a state of high glucose, autophagy is inhibited, resulting in impaired islet function, insulin resistance, and complications [129]. In AD, defects in endocytosis and autophagy have emerged as key drivers of the accumulation of cellular waste products, progressive neuronal dysfunction, and neurodegeneration [130]. A systematic review and meta-analysis suggested that the dysregulation of proteins in the endosomal–lysosomal and autophagy pathway may play an important role in AD pathogenesis [131].

The overexpression and overactivity of GSK-3 in the skeletal muscle of rodent models of obesity and obese type 2 diabetic humans are associated with the impaired ability of insulin to activate glucose disposal and glycogen synthase [132]. The selective inhibition of GSK-3 in insulin-resistant skeletal muscle causes improvements in insulin-stimulated glucose transport activity that are likely caused by enhanced post-insulin receptor insulin signaling and GLUT4 translocation [132]. Inhibiting GSK-3 activity by pharmacological intervention has become an important strategy for the management of type 2 diabetes [133]. The inhibition of GSK-3 improves insulin signaling through the IRS-1, PI3K, and protein kinase B (PKB/Akt) pathways [133]. GSK-3 overactivation disrupts neural growth, development, and function. It directly promotes tau phosphorylation and regulates APP cleavage, leading to Aβ formation, and it directly or indirectly triggers neuroinflammation and oxidative damage [134].

The islet amyloid polypeptide (IAPP) plays a role in glucose homeostasis but aggregates to form islet amyloid in type 2 diabetes [135]. Islet amyloid formation contributes to β-cell dysfunction and death. IAPP is associated with the activation of the inflammasome, defects in autophagy, endoplasmic reticulum (ER) stress, the generation of ROS, and membrane disruption in AD brains. AD and type 2 diabetes are considered protein misfolding disorders associated with the accumulation of protein aggregates, namely Aβ and tau in the brain during AD and IAPP in pancreatic islets in type 2 diabetes. Misfolded IAPP produced in type 2 diabetes may potentiate AD pathology by cross-seeding Aβ, providing a molecular explanation for the link between these diseases [136].

**Figure 3:**
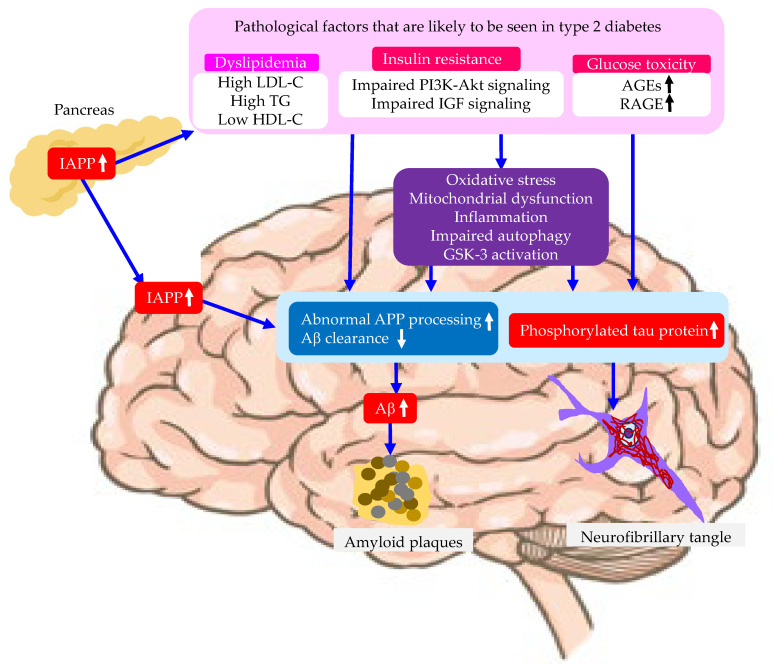
Risk factors related with type 2 diabetes for the development of AD. Aβ, amyloid beta; AGEs, advanced glycation end products; APP, amyloid precursor protein; GSK-3, glycogen synthase kinase-3; HDL-C, high-density lipoprotein cholesterol; IAPP, islet amyloid polypeptide; IGF, insulin-like growth factor; LDL-C, low-density lipoprotein cholesterol; PI3K, phosphatidyl-inositide 3-kinases; RAGE, receptor for AGEs; TG, triglyceride.

In summary, risk factors related with type 2 diabetes for the development of AD are shown in Figure 3. Insulin resistance, hyperglycemia, and diabetic dyslipidemia, which are commonly observed in type 2 diabetes, may directly induce brain pathological changes in AD patients or induce oxidative stress, mitochondrial dysfunction, inflammation, impaired autophagy, and GSK-3 activation. IAPP influences the development and progression of both type 2 diabetes and AD.

## 5. Changes in Energy Metabolism by Type 2 Diabetes and AD

GLUT1 is expressed in all brain cells, including endothelial cells, and with very low neuronal expression, while GLUT3 is almost restricted to neurons [137]. Levels of the main BBB carrier GLUT1 were found to be reduced in the hippocampus of insulin-resistant rats [138]. Lower levels of both GLUT1 and GLUT3 were found in the brains of mice under a diet rich in fat and sugar for 3 months [139]. Mice fed a high-fat diet for 3 months also showed reduced density of neuronal GLUT3 and of insulin-dependent GLUT4, which is key for synaptic fueling, when compared to controls [140]. Hippocampal neurons of obese type 2 diabetic subjects displayed reduced GLUT4 expression and neuronal soma area, associated with an increased expression of NF-κB. [141]. Insulin resistance and type 2 diabetes reduce GLUT3 and GLUT4, which are crucial GLUTs for neurons. Such reduced expressions of GLUT1, GLUT3, and GLUT4 may induce cerebral glucose hypometabolism, which is characteristic of AD. Post-mortem studies showed consistent reductions in GLUT1 and GLUT3 in the hippocampus and cortex of AD brains [142]. Uncontrolled microglial activation has been implicated in the development of AD [143], and glucose uptake in microglia is facilitated predominately by GLUT1 under inflammatory conditions [53]. AD patients exhibited a positive association between glucose uptake and microglial activity [144]. GLUT2 was significantly increased in AD brains and the brains of subjects with both AD and type 2 diabetes, possibly due to astrocyte overactivation [145]. Thus, the increase in GLUT2 level in AD brain homogenates is most likely due to astrocyte overactivation, which is a well-known phenomenon in AD brains [146]. Increased glucose uptake by activated microglia may deprive glucose availability in neurons, astrocytes, and oligodendrocytes. Oligomeric Aβ causes impaired hippocampal insulin signaling and reduced GLUT4 translocation, accompanied by cognitive impairment and hippocampal hypometabolism [147]. GLUT4 immunoreactivity colocalizes with cholinergic markers [148], suggesting a role for GLUT4 dysregulation in AD, which is characterized by damage to cholinergic neurons [149,150].

Astrocytes mainly express MCT1 and MCT4, whereas neurons mainly express MCT2 [151,152]. The activity of the astrocyte–neuron lactate shuttle is involved in brain energetic support, and the process of lactate transport from astrocytes to neurons, which is important in neuronal energy support, depends on the activity of MCTs [153,154]. MCT2 protein levels in type 2 diabetes model rats decreased significantly in the hippocampus and hypothalamus compared to their controls [155], suggesting that type 2 diabetes reduces lactate or ketone bodies’ availability in neurons. Alterations to cerebral lactate metabolism in the double-transgenic APP/presenilin 1 (APP/PS1) mouse model of AD were studied [156]. Lactate content and the expressions of cerebral MCT1, MCT2, and MCT4 were decreased in APP/PS1 mice, suggesting that decreases in cerebral lactate content and MCTs may lead to the blockage of lactate and ketone body transport from glia to neurons, resulting in neuronal energy substrate deficit in neurons.

Although FA utilization is less preferred in neurons [54], neurons in AD patients cannot use glucose, lactate, and ketone bodies due to the reduced expression of GLUTs and MCTs. Therefore, neurons in AD patients must use FAs. Serum brain-type FABP levels were significantly elevated in AD patients by 29% as compared with healthy donors (2%) [157]. CSF levels of FABP3 were elevated in AD patients compared with control subjects (*p* < 0.01) [158]. Another study showed that CSF FABP3 concentration distinguished between healthy controls and patients with AD with a sensitivity and specificity of 76% and 84%, respectively. Both patients with AD and MCI due to AD had higher FABP3 levels in CSF when compared with cognitively healthy controls [159]. Increased FA use in the brain may be a crucial determinant of the development of AD.

Accumulated FFAs are metabolized into ceramides [160]. The excessive accumulation of ceramides induces cellular stress responses, leading to apoptosis and contributing to the development of type 2 diabetes and AD [161,162,163]. Ceramide accumulation in the brain may be directly caused by increased FFA levels or indirectly promoted by FFA-induced neuroinflammation [164,165]. An increase in ceramide was observed in the brains of early-stage AD patients [166]. Healthy microglia can remove ceramide via scavenger receptors [81]. However, activated microglia cannot clear ceramide, which may increase ceramide accumulation.

Astrocytes provide key neuronal support, and their phenotypic transformation is implicated in neurodegenerative diseases. The brain critically depends on astrocytic mitochondrial oxidative phosphorylation to degrade FAs. Astrocytic mitochondrial dysfunction is observed in patients with type 2 diabetes [167]. Aberrant astrocytic mitochondrial oxidative phosphorylation induces lipid droplet accumulation followed by neurodegeneration [168]. When the FA load overwhelms astrocytic mitochondrial oxidative phosphorylation capacity, elevated acetyl-CoA levels induce astrocyte reactivity by enhancing signal transducer and activator of transcription 3 acetylation and activation [168]. Intercellularly, lipid-laden reactive astrocytes stimulate neuronal FA oxidation and oxidative stress, activate microglia through interleukin-3 signaling, and inhibit the biosynthesis of FAs and phospholipids required for myelin replenishment [168].

Microglia become increasingly dysfunctional with aging and contribute to the onset of AD through defective phagocytosis, attenuated cholesterol efflux, and excessive secretion of pro-inflammatory cytokines. High blood glucose drives microglial activation and M1 polarization, and M1 microglia release pro-inflammatory cytokines, causing neuronal damage [169]. Dysfunctional microglia also accumulate lipid droplets. Microglia lacking LPL showed excessive accumulation of lipid droplet-like structures and displayed a pro-inflammatory lipidomic profile, increased cholesterol ester content, and reduced cholesterol efflux [170]. Peroxisome proliferator-activated receptor (PPAR) agonists rescued the lipid droplet-associated phenotype in microglia lacking LPL [170].

Heurling, K. et al. also showed the overlap between type 2 diabetes and AD by using medical imaging, focusing on glucose metabolism, mitochondrial function, and lipid metabolism [171]. Dewanjee, S. et al. systematically assessed abnormal glucose metabolism associated with Ab and phosphorylated tau accumulation in AD brains, and they suggested the emerging role of altered glucose metabolism in contributing to the impact of insulin signaling and mitochondrial dysfunction in defective cerebral glucose utilization in AD [172].

In summary, changes in the expression of GLUTs by type 2 diabetes and AD are shown in Table 2. The changes in GLUT expression induced by type 2 diabetes agree with the changes in GLUT expression observed in AD, suggesting that the altered expression of GLUTs is associated with the development of AD.

**Figure 4:**
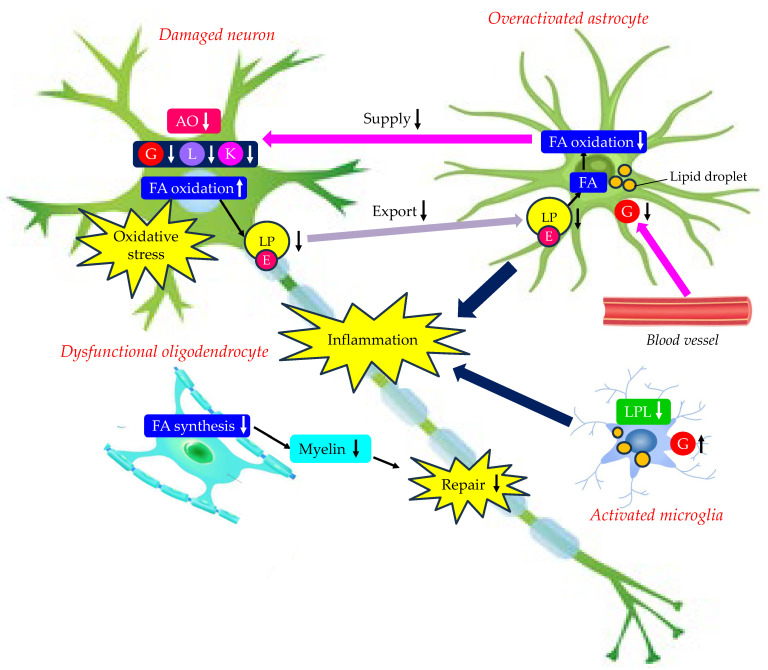
Effects of changes in energy metabolism in brain due to type 2 diabetes on the development of AD. AO, antioxidant; E, apo E; FA, fatty acid; G, glucose; K, ketone body; L, lactate; LP, lipoprotein-like particle; LPL, lipoprotein lipase. Upward and downward arrows indicate an increase and decrease in expression, activity, and phenomenon, respectively.

The effects of changes in energy metabolism in the brain due to type 2 diabetes on the development of AD are shown in Figure 4. Neurons are provided with energy substrates by astrocytes. Insulin resistance and type 2 diabetes may reduce GLUT1 expression in BBB, neurons, astrocytes, and oligodendrocytes and also reduce GLUT3 and 4 expression in neurons. Increased glucose uptake by activated microglia further deprives glucose availability by neurons. Dysfunctional astrocytes cannot supply glucose, lactate, and antioxidants to neurons. Astrocytic dysfunctional mitochondria cannot oxidize FA, resulting in a reduced supply of ketone bodies to neurons. Neurons critically depend on astrocytic mitochondrial oxidation to degrade FAs. Dysfunctional over-activated astrocytes stimulate neuronal FA oxidation and increase neuronal oxidative stress, activate microglia, and inhibit the biosynthesis of FAs required for myelin replenishment in oligodendrocytes. Furthermore, activated astrocytes and microglia induce inflammation. Such phenotypic changes in neurons, astrocytes, microglia, and oligodendrocytes due to changes in energy metabolism by type 2 diabetes may be associated with the development of AD.

## 6. Possible Therapeutic Intervention for AD Considering AD Risk Factors

### 6.1. Exercise

Exercise exerts neuroprotective effects by ameliorating mitochondrial dysfunction in the neurons of AD [173], which involves multiple mechanisms, including mitochondrial dynamics, biogenesis, mitophagy, transport, and signal transduction [173,174]. A meta-analysis, including 29 prospective cohort studies, showed a favorable effect of physical activity on AD risk decline (HR, 0.72; 95% CI, 0.65 to 0.80). A subgroup analysis of physical activity intensity demonstrated an inverse dose–response relationship between physical activity and AD, the effect sizes of which were significant in moderate (HR, 0.85; 95% CI, 0.80 to 0.93) and high physical activity (HR, 0.56; 95% CI, 0.45 to 0.68) but not in low physical activity (HR, 0.94; 95% CI, 0.77 to 1.15) [175]. A meta-analysis, including 27 randomized controlled trials (RCTs), showed a nonlinear dose–response relationship between exercise and cognitive improvement in AD, with the optimal aerobic exercise dose identified at 660 METs-min/week for enhancing cognitive function in AD [176].

### 6.2. Antioxidants

#### 6.2.1. Vitamin E

Experimental models showed that vitamin E supplementation, by decreasing oxidative stress, may be a good strategy for improving cognitive and memory deficits in AD [177]. Individuals with AD had lower circulatory concentrations of α-tocophenol compared with healthy controls [178]. Another meta-analysis also showed that AD patients had a lower concentration of serum vitamin E compared with healthy controls among older people (weighed mean difference (WMD), −6.811 μmol/L; 95% CI, −8.998 to −4.625; *p* < 0.001) [179]. A meta-analysis using observational studies showed that high vitamin E intake from diet and supplements significantly reduced the risk of AD (odds ratio (OR), 0.78; 95% CI, 0.64 to 0.94) [180].

#### 6.2.2. Vitamin C

Vitamin C is an excellent water-soluble antioxidant, is capable of donating electrons to neutralize free radicals, and supports the regeneration of other antioxidants [181,182]. The CSF vitamin C concentration is about 3–4 times higher than plasma [183] and about 200 times higher in neurons compared with plasma [184]. Vitamin C levels were measured in the CSF and plasma of 10 AD patients and 10 controls [185]. The daily dosage of vitamin C was significantly correlated with both plasma and CSF vitamin C levels. In AD patients, hippocampal volume was significantly correlated with plasma and CSF vitamin C levels. A quantitative meta-analysis of vitamin C in the pathophysiology of AD using observational studies showed that deficiency in vitamin C is involved in AD progression, and supplementation is a plausible preventive and AD treatment strategy [186]. However, the necessary vitamin C levels to prevent the development of AD is unknown.

#### 6.2.3. Resveratrol

Resveratrol is a potent Sirtuin1 enhancer that facilitates neuroprotection and promotes neurogenesis in the hippocampus of the adult brain [187]. In a meta-analysis comprising five trials involving 271 AD patients, compared with placebo therapy, resveratrol treatment resulted in a significant improvement in AD (Cooperative Study–Activities of Daily Living scores, CSF Aβ40 and plasma Aβ40 levels (standard mean difference (SMD), 0.43; 95% CI, 0.07 to 0.79) [188].

### 6.3. Statins

Statins are known to decrease cholesterol levels in the brain membranes and further exhibit anti-inflammatory, antioxidant, and neuroprotective properties [189]. Preclinical and observational studies have suggested that statins may offer protective benefits against the development of AD [190,191,192]. RCTs using simvastatin and atorvastatin have not confirmed the beneficial effects of statins in AD patients [193,194]. Recent research has suggested that statin could be particularly advantageous for individuals with the ApoE4 genotype [189].

### 6.4. PPARa Agonists

Bezafibrate, a PPAR pan-agonist, has been shown to rescue mitochondrial dysfunction and to significantly improve cognitive/memory function in AD mice accompanied by the alleviation of amyloid pathology and neuronal loss as well as reduced oxidative stress and neuroinflammation [195]. Fenofibrate increased the expression of PPARα and reduced soluble APP and Aβ released in APP/PS1 transgenic mice [196]. At present, no RCT has been conducted to evaluate the usefulness of PPARa agonists for AD patients.

### 6.5. N-3-Polyunsaturated Fatty Acids (PUFAs)

N-3 PUFAs, such as eicosatetraenoic acid (EPA) and docosahexaenoic acid (DHA), are essential nutrients obtained from the diet, usually found in fatty fish and fish oil supplements. They are essential for brain cellular membranes, and DHA has been shown to be especially essential in normal neuronal development [197]. The brain contains large amounts of n-3 PUFAs, predominantly DHA, suggesting functional brain changes with n-3 PUFA deprivation [198]. EPA has significant anti-inflammatory effects by inhibiting cytokine release from leukocytes [199]. In a systematic review and meta-analysis including 11 RCTs and 3 observational studies, the Clinical Dementia Rating scale showed reduced progression of cognitive decline among patients with n-3 PUFA supplemental interventions, with no differences between different n-3 PUFA supplements [200].

### 6.6. Anti-Diabetic Drugs

A systematic umbrella review and meta-analysis including 100 reviews and 27 cohort/case–control studies (*n* = 3,046,661) showed that metformin, pioglitazone, glucagon-like peptide 1 receptor agonists (GLP-1RAs), and sodium glucose co-transporter-2 inhibitors (SGLT2is) were associated with a significant reduction in the risk of dementia [201]. This indicates that anti-diabetic drugs that improve insulin resistance are associated with a reduced risk of developing AD.

#### 6.6.1. Pioglitazone

Pioglitazone, a PPARγ agonist, improves insulin resistance and is known to have anti-inflammatory and antioxidant effects on the brain, and its clinical potential in the treatment of cognitive impairment in diseases such as AD is currently being explored [202]. Pioglitazone promotes the function of PPAR receptors in ameliorating inflammation, oxidative stress, amyloidogenesis, and enhancing neurogenesis, synaptic plasticity, and mitochondrial function [202]. The TOMORROW study, a phase III RCT to evaluate the safety and efficacy of low-dose pioglitazone (0.8 mg/day sustained release) in delaying the onset of mild cognitive impairment in participants at a high risk for AD, showed that pioglitazone did not delay the onset of mild cognitive impairment [203]. Regarding this trial, the question is whether the daily dose of pioglitazone was appropriate.

#### 6.6.2. Metformin

Metformin improves insulin resistance. Further, experimental studies have provided proof-of-concept for metformin, including a study in which it stimulated the microglial-induced phagocytosis of amyloid deposits and tau proteins, thereby reducing amyloidogenesis in a mouse model [204]. However, a meta-analysis of 10 observational studies did not support a reduction in the risk of AD with metformin [205]. Three relevant clinical trials, namely Metformin in Alzheimer’s Dementia Prevention (MAP) (NCT04098666), MET-FINGER (NCT05109169), and MET-MEMORY (NCT04511416), are currently underway based on the above experimental proof of concept; each is expected to report in 2027 [206].

#### 6.6.3. SGLT2is

SGLT2is has multifaceted mechanisms of action for the treatments of AD, such as encompassing antioxidative stress, anti-neuroinflammation, the upregulation of autophagy, anti-apoptosis, the protection of endothelial cells, and acetylcholinesterase (AChE) inhibitor activity [207]. Acetylcholine is one of the important neurotransmitters in the human brain and participates in the electrophysiological activity between neurons, ensuring signal transmission between neurons [208]. The cholinergic hypothesis revealed the important role of acetylcholine in AD, emphasizing that synaptic loss and atrophy in AD impair neurotransmitter conduction [209]. The reduced binding of acetylcholine and cholinergic receptors is one of the important reasons for the emergence of psychiatric symptoms in AD patients. Therefore, AChE inhibitors (such as donepezil and galantamine) are used to improve cognitive function in AD patients. SGLT2 inhibition significantly reduced AChE activity and increased monoamine levels, improving memory dysfunction in mice [210]. There are emerging data from murine studies that SGLT2 is can cross the BBB and may have neuroprotective effects, such as increasing the brain-derived neurotrophic factor, reducing the amyloid burden, and inhibiting AChE [211].

A very recent database containing information on medications prescribed to 233,183 patients aged 50 years or older between 2018 and 2020 showed that GLP-1RAs and SGLT2is might be associated with lower odds of anti-AD drug usage [212]. A retrospective examination of data from a cohort of 1,348,362 participants with type 2 diabetes (≥40 years) showed that SGLT2is use was associated with a reduced risk of AD (HR, 0.81; 95% CI, 0.76 to 0.87) [213]. No RCT has ever been conducted to evaluate the usefulness of SGLT2is for AD.

#### 6.6.4. GLP-1RAs

GLP-1RAs have been demonstrated to have significant benefits in models of neurodegenerative diseases by modulating a variety of pathogenic mechanisms, including neuroinflammation, autophagy, mitochondrial dysfunction, and the abnormal phosphorylation of pathognomonic proteins [214]. In an AD mouse model featuring APP/PS1 mice, liraglutide prevented memory impairments in object recognition and water maze tasks and prevented synapse loss and the deterioration of synaptic plasticity in the hippocampus [215]. In liraglutide-treated APP/PS1 mice, the overall β-amyloid plaque count in the cortex and dense-core plaque numbers were reduced by 40–50%, while levels of soluble amyloid oligomers were reduced by 25%. Activated microglia were significantly reduced by liraglutide. Liraglutide increased young neurons in the dentate gyrus in APP/PS1 mice. A systematic review and meta-analysis of preclinical studies showed that GLP-1RAs could improve the learning and memory abilities of AD rodents; in terms of pathology, GLP-1 Ras could reduce Aβ deposition and phosphorylated tau levels in the brains of AD rodents [216].

Sirtuin1 was found to be closely related to the expression of GLP-1R in the hippocampus of AD model mice [217]. Semaglutide increased the expression levels of Sirtuin1 and GLUT4 in the hippocampus of AD model mice, accompanied by an improvement in learning and memory and a decrease in Aβ plaques and neurofibrillary tangles [217]. Further, semaglutide improved glucose metabolism by promoting glycolysis, improving glycolytic disorders, and increasing the membrane translocation of GLUT4 in the brain of AD model mice. These effects were blocked by the Sirtuin1 inhibitor, suggesting that Sirtuin1/GLUT4 signaling pathway may be an important mechanism for GLP-1RAs to promote glucose metabolism in the brain. Two phase III clinical trials testing semaglutide (Wegovy, Ozempic, Rybelsus) in AD patients are underway (NCT04777396 and NCT04777409) [218].

#### 6.6.5. Dual GLP-1 and Glucose-Dependent Insulinotropic Polypeptide (GIP) Receptor Agonist (Dual GLP-1/GIP RA)

GIP is a member of the incretin hormones and growth factors. Neurons express the GIP receptor, and GIP and its agonists can pass through the BBB and show remarkable neuroprotective effects by protecting synapse function and numbers, promoting neuronal proliferation, reducing amyloid plaques in the cortex, and reducing inflammation [219]. Dual GLP-1/GIP RA administration significantly prevented spatial learning deficits and decreased phosphorylated tau levels in the rat cerebral cortex and hippocampus in streptozotocin-induced AD model rats [220]. Dual GLP-1/GIP RA reduced chronic inflammation and apoptosis by reactivating insulin signaling pathways. Dual GLP-1/GIP RA improved cognitive impairment in a range of tests and relieved the pathological features of APP/PS1/tau mice, enhanced long-term potentiation in the hippocampus, increased the number of synapses and dendritic spines, and normalized the volume and number of mitochondria while downregulating amyloids and phosphorylated tau protein [221]. Dual GLP-1/GIP RA was more effective in reversing memory loss, enhancing synaptic plasticity in the hippocampus, reducing amyloid plaques, and lowering pro-inflammatory cytokine levels in the brain than liraglutide in the APP/PS1 mouse model of AD [222]. To date, no RCTs or observational studies have studied the usefulness of dual GLP-1/GIP RA for AD patients.

#### 6.6.6. Imeglimin

Mitochondrial dysfunction is a prominent pathological feature of type 2 diabetes and AD. Apoptotic cell death has been shown to constitute the terminal process in AD. A decrease in mitochondrial membrane potential causing the opening of the permeability transition pore (PTP) in mitochondrial membranes has been implicated as a critical effector of apoptosis [223]. The opening of the PTP leads to the release of so-called apoptosis initiation factors that induce the degradative events of apoptosis [223].

Imeglimin reduces ROS production, improves mitochondrial function and integrity, and results in enhancing glucose-stimulated insulin secretion and inhibiting the apoptosis of β-cells [224]. Imeglimin has been reported to significantly reduce the size of cerebral infarction, cerebral edema, and neurological defects due to cerebral ischemia in rats [225]. Imeglimin reduced ischemia-induced neuronal loss by preventing microglial activation and by increasing astrocytes that produce anti-inflammatory cytokines [225]. Imeglimin acutely prevented the opening of the PTP in neurons and astrocytes, which may contribute to the reduced apoptosis of such cells. To date, no RCTs or observational studies have studied the usefulness of imeglimin for AD patients.

## 7. Conclusions and Future Directions

To our knowledge, this article is the first to comprehensively describe cooperation in energy metabolism and the removal of FA toxicity between normal brain cells, the influence of changes in such cooperation due to type 2 diabetes on AD development, and the possible therapeutic interventions for unfavorable changes due to type 2 diabetes and AD.

The risk factors related to type 2 diabetes for the development of AD include insulin resistance, hyperglycemia, diabetic dyslipidemia, oxidative stress, mitochondrial dysfunction, inflammation, impaired autophagy, and GSK-3 activation. Such risk factors for AD are mutually associated with harmful phenotypic changes in neurons, astrocytes, microglia, and oligodendrocytes. Neurons are provided with energy by astrocytes. Insulin resistance and type 2 diabetes may reduce GLUT1 expression in the BBB, neurons, astrocytes, and oligodendrocytes and also reduce GLUT3 and 4 expression in neurons. Increased glucose uptake by activated microglia further deprives glucose availability in neurons. Dysfunctional astrocytes cannot supply glucose, lactate, and antioxidants to neurons. Astrocytic dysfunctional mitochondria cannot oxidize FAs, resulting in a reduced supply of ketone bodies to neurons. Neurons critically depend on astrocytic mitochondrial oxidation to degrade FAs. Overactivated astrocytes stimulate neuronal FA oxidation and increase neuronal oxidative stress, activate microglia, and inhibit the biosynthesis of FAs required for myelin replenishment in oligodendrocytes. Furthermore, activated astrocytes and microglia induce inflammation.

Neuroprotective effects, effects on energy metabolism in AD brain, clinical studies, and future directions for application to AD treatment or possible therapeutic interventions for AD are shown in Table 3.

## Figures and Tables

**Table 1:** Glucose transporter (GLUT) and monocarboxylate transporter (MCT) in brain cells and blood–brain barrier.

Brain Cells	GLUTs	MCTs
Neuron	GLUT1	MCT2
	GLUT3	
	GLUT4	
Astrocyte	GLUT1	MCT1
	GLUT2 ↑ (overactivation)	MCT4
Oligodendrocyte	GLUT1	MCT1
Microglia	GLUT3	MCT1
	GLUT5	MCT2
	GLUT1 ↑ (inflammation)	MCT4
Blood–brain barrier		
	GLUT1	MCT1

Upward arrows (↑) indicate an increase in expression.

**Table 2:** Changes in expressions of glucose transporters in the brain by type 2 diabetes and AD.

Glucose Transporters	Type 2 Diabetes	AD
GLUT1	↓	↓ (↑ in microglia)
GLUT2	↑ (in astrocytes)	↑ (in astrocytes)
GLUT3	↓	↓
GLUT4	↓	↓

Upward (↑) and downward arrows (↓) indicate an increase and decrease in expression, respectively.

**Table 3:** Neuroprotective effects, effects on energy metabolism in AD brain, clinical studies, and future directions for application to AD treatment or possible therapeutic interventions for AD.

Therapeutic Interventions	Neuroprotective Effects	Effects on Energy Metabolism in AD Brain	Clinical Studies	Future Directions for Application to AD Treatment
Exercise	An improvement in mitochondrial dysfunction	An improvement in glucose/lipid metabolism and insulin signaling	The meta-analysis, including 27 RCTs, showed a non-linear dose–response relationship between exercise and cognitive improvement in AD, with the optimal aerobic exercise dose identified at 660 METs-min/week for enhancing cognitive function in AD [176].	It is necessary to devise an exercise protocol that can be continued by many people and suppress the onset of AD and to examine the effectiveness of combining exercise with antioxidant vitamins.
Vitamin E	Reduction of oxidative stress	An improvement in glucose/lipid metabolism and insulin signaling	The meta-analysis, using observational studies, showed that high vitamin E intake significantly reduced the risk of AD [180].	It is necessary to examine the daily dose that is effective in suppressing the development of AD and to conduct RCTs using such doses of vitamin E.
Vitamin C	Reduction in oxidative stress	An improvement in glucose/lipid metabolism and insulin signaling	A meta-analysis using observational studies showed that a deficiency in vitamin C is involved in AD progression, and supplementation is a plausible preventive and AD treatment strategy [186].	It is necessary to examine the daily dose that is effective in suppressing the development of AD and to conduct RCTs using such doses of vitamin E.
Resveratrol	Reduction in oxidative stress and enhancement of Sirtuin1	An improvement in glucose/lipid metabolism and insulin signaling	The meta-analysis comprised 5 trials involving 271 AD patients, and compared with placebo therapy, resveratrol treatment resulted in a significant improvement in AD symptoms and Aβ accumulation [188].	It is necessary to determine the daily dose that is effective in suppressing the development of AD and to conduct RCTs with more AD patients.
Statins	Reduction in cholesterol and anti-inflammatory and antioxidant effects	An improvement in lipid metabolism and insulin signaling	Preclinical and observational studies have suggested that statins may offer protective benefits against the development of AD [190,191,192]. RCTs using statins have not confirmed the beneficial effects of statins in AD patients [193,194].	It is necessary to consider appropriate doses of statins other than simvastatin and atorvastatin that are effective in suppressing the development of AD and to conduct RCTs using such statins.
PPARa agonists	An improvement in mitochondrial dysfunction and insulin resistance	An improvement in glucose/lipid metabolism and insulin signaling	Bezafibrate significantly improved cognitive/memory function in AD mice [195]. Fenofibrate reduced soluble APP and Aβ releases in APP/PS1 transgenic mice [196].	RCT should be conducted to evaluate the usefulness of PPARa agonists for AD patients.
N3-PUFA	Beneficial for neuronal development and anti-inflammatory effect	An improvement in lipid metabolism	In the meta-analysis, including 11 RCTs and 3 observational studies, the reduced progression of cognitive decline among patients with n-3 PUFA supplemental interventions was observed [200].	It is necessary to determine the daily minimal dose that is effective in suppressing the development of AD.
Pioglitazone	An improvement in insulin resistance; ameliorating inflammation, oxidative stress, and amyloidogenesis; and enhancing neurogenesis, synaptic plasticity, and mitochondrial function	An improvement in glucose/lipid metabolism and insulin signaling	The TOMORROW study, a phase III RCT, showed that low-dose pioglitazone (0.8 mg/day sustained release) did not delay the onset of mild cognitive impairment [203].	The dose used in the TOMORROW study was very low, and RCTs using higher doses of pioglitazone should be performed.
Metformin	An improvement in insulin resistance and the stimulation of microglial-induced phagocytosis of amyloid deposits and tau proteins	An improvement in glucose/lipid metabolism and insulin signaling	The meta-analysis of 10 observational studies did not support a reduction in the risk of AD with metformin [205]. Three clinical trials are currently underway [206].	The results of ongoing RCTs should be evaluated and verified, and the effectiveness of metformin for AD should be accurately assessed.
SGLT2is	An improvement in insulin resistance and anti-inflammatory and antioxidant effects, the upregulation of autophagy, anti-apoptosis, and the protection of endothelial cells, as well as the inhibition of acetylcholinesterase	An improvement in glucose/lipid metabolism and insulin signaling	A retrospective examination of data from a cohort of 1,348,362 participants with type 2 diabetes showed that SGLT2is use was associated with a reduced risk of AD [213].	No RCT has ever been conducted to evaluate the usefulness of SGLT2is for AD. RCTs using SGLT2is should be performed.
GLP-1RAs	An improvement in mitochondrial dysfunction, inflammation, and autophagy, as well as the prevention of synapse loss	An improvement in glucose/lipid metabolism and insulin signaling	The meta-analysis of preclinical studies showed that GLP-1 RAs could improve the learning and memory abilities of AD rodents; GLP-1 RAs could reduce Aβ deposition and phosphorylated tau levels in the brains of AD rodents [216]. Two phase III clinical trials testing semaglutide in AD patients are underway [218].	The results of ongoing RCTs should be evaluated and verified, and the effectiveness of GLP-1RAs for AD should be accurately assessed.
Dual GLP-1/GIP RA	The protection of synapses, the promotion of neuronal proliferation, and an improvement in mitochondrial dysfunction	An improvement in glucose/lipid metabolism and insulin signaling	Dual GLP-1/GIP RA was more effective in reversing memory loss, enhancing synaptic plasticity in the hippocampus, reducing amyloid plaques, and lowering pro-inflammatory cytokine levels in the brain than liraglutide in an APP/PS1 mouse model of AD [222].	No RCTs or observational studies have investigated the usefulness of dual GLP-1/GIP RA for AD patients. RCTs using dual GLP-1/GIP RA should be performed.
Imeglimin	An improvement in mitochondrial dysfunction	An improvement in glucose/lipid metabolism and insulin signaling	Imeglimin reduced ischemia-induced neuronal loss, by preventing microglial activation, and by increasing astrocytes that produce anti-inflammatory cytokines in rats [225].	No RCTs or observational studies have investigated the usefulness of imeglimin for AD patients. RCTs using imeglimin should be performed.

AD, Alzheimer’s disease; GIP, glucose-dependent insulinotropic polypeptide; GLP-1RAs, glucagon-like peptide 1 receptor agonists; HDL-C, high-density lipoprotein cholesterol; LDL-C, low-density lipoprotein cholesterol; PPAR, peroxisome proliferator-activated receptor; SGLT2is, sodium-glucose co-transporter-2 inhibitors; TG, triglyceride.

## Abbreviations

Aβ	amyloid beta
AChE	acetylcholinesterase
AD	Alzheimer’s disease
AGEs	advanced glycation end products
APP	amyloid precursor protein
BBB	blood–brain barrier
CI	confidence interval
CNS	central nervous system
CSF	cerebrospinal fluid
DHA	docosahexaenoic acid
EPA	eicosatetraenoic acid
ER	endoplasmic reticulum
FA	fatty acid
FABP	fatty acid binding protein
FATP	fatty acid transport proteins
GIP	glucose-dependent insulinotropic polypeptide
GLP-1RAs	glucagon-like peptide 1 receptor agonists
GLUT	glucose transporter
GSK-3	glycogen synthase kinase-3
HDL-C	high-density lipoprotein-C
HOMA-IR	homeostatic model assessment of insulin resistance
HR	hazard ratio
IAPP	islet amyloid polypeptide
IGF1	insulin-like growth factor 1
IR	insulin receptor
IRS	insulin receptor substrate
LDL-C	low-density lipoprotein cholesterol
LPL	lipoprotein lipase
MAPK	mitogen-activated protein kinase
MCT	monocarboxylate transporter
MMSE	Mini-Mental State Examination scale score
MRI	magnetic resonance imaging
OR	odds ratio
PET	positron emission tomography
PI3K	phosphatidyl-inositide 3-kinase
PPAR	peroxisome proliferator-activated receptor
PS1	presenilin 1
PTP	permeability transition pore
PUFA	polyunsaturated fatty acid
RAGE	Receptor for advanced glycation end products
RCT	randomized controlled trial
ROS	reactive oxygen species
RR	relative risk
SGLT2is	sodium glucose co-transporter-2 inhibitors
SMD	standard mean difference
WMD	weighed mean difference
